# Mesenchymal stem cells in alleviating sepsis-induced mice cardiac dysfunction via inhibition of mTORC1-p70S6K signal pathway

**DOI:** 10.1038/cddiscovery.2016.97

**Published:** 2017-02-27

**Authors:** Wei Huang, Wensi Fan, Yabin Wang, Dong Han, Xiujuan Li, Shuang Li, Congye Li, Bin Xu, Yuesheng Huang, Xiaobin Fu, Feng Cao

**Affiliations:** 1 Department of Cardiology, Chinese PLA General Hospital, Beijing, China; 2 Department of Cardiology, Xijing Hospital, Fourth Military Medical University, Shanxi, China; 3 Institute of Burn Research, State Key Laboratory of Trauma, Burns and Combined Injury, Southwest Hospital, The Third Military Medical University, Chongqing, China

## Abstract

Sepsis-induced cardiac dysfunction remains a major cause of morbidity and mortality in patients suffered from severe trauma. Mesenchymal stem cells (MSCs) -based treatment has been verified as a promising approach to mitigate the sepsis-induced cardiac dysfunction, but the mechanism is still ambiguous. Thus, our study was designed to explore the potential role of MSCs in sepsis-induced cardiac dysfunction. *In vivo* bioluminescence imaging revealed 80% acute donor cell death of bone marrow-derived MSCs (BM-MSCs) within 3 days after transplantation. However, echocardiography demonstrated that systolic function in wild-type mice group were reduced after sepsis, while the cardiac function was relatively well persevered in cardiac-conditional deletion of Raptor (component of mTORC1 complex) mice group. Raptor KO group treated with BM-MSCs appeared better cardiac function than other groups (*P*<0.05). *In vitro* cell study revealed that co-culture of H9C2 (Raptor-Knock down) and BM-MSC could attenuate the level of proinflammatory cytokines and promote the expression of anti-inflammatory cytokine accompanied by mTORC2-Akt activation (*P*<0.05). In contrast, co-culture H9C2 (Raptor-O.E) and BM-MSC could aggravate the inflammatory response accompanied by the activation of mTORC1-p70S6K and inhibition of mTORC2-Akt (*P*<0.05). The immunomodulatory property of MSC is related to the inhibition of mTORC1-p70S6K and activation of mTORC2-Akt signaling pathway. mTORC1-p70S6K and mTORC2-Akt pathways were involved in the therapeutic adjuncts of MSC. The possible mechanism due to MSC`s immunomodulatory property through activation of mTORC2-Akt and inhibition of mTORC1-p70S6K signal pathways which may lead to modulate the expression of inflammation cytokines.

## Introduction

Sepsis is a lethal clinical disease causing severe multiple organ dysfunction syndrome.^1^ Although the prognosis of sepsis has improved, the mortality still remains high.^2^ Myocardium and cardiomyocytes are undermined in this pathological process.^3,4^ Sepsis-induced cardiomyopathy (SIC) is thought to be the consequence of circulating pathological factors, such as bacterial lipopolysaccharide, inflammatory cytokines.^5,6^ Although antibiotic, fluid resuscitation and general management are applied in sepsis therapy, irreversible cardiomyocytes death and decline of cardiac function have already occurred.^7,8^

In sepsis, excessive inflammation and deregulation of the immune system are important detrimental effects on the host.^9,10^ A few recent reports and our previous studies has demonstrated that application of mesenchymal stem cells (MSCs) provides a promising approach for tissue regeneration, functional recovery and immunomodulation in sepsis, peripheral arterial and cardiovascular disease.^11–14^ MSCs may exhibit immunosuppressive or immunomodulatory properties and they can be easily obtained and isolated from the bone marrow^15,16^ However, the mechanism of MSC treatment is still unknown. Furthermore, noninvasive cell monitoring technique is reported to be useful in evaluating the longitudinal survival and behavior of the donor cells *in vivo*.^17^

The mechanistic target of rapamycin (mTOR) is a ubiquitous serine/threonine kinase, which was recently identified to modulate proinflammatory effects in activation status. mTOR assembles into two complexes: mTOR complex 1 (mTORC1) and complex 2 (mTORC2). mTORC1 contains the distinct partner protein (for example, regulatory-associated protein of mTOR Raptor), which are proven to orchestrate various cues including inflammation, survival and apoptosis.^18,19^ Several studies showed that mTOR was involved in LPS-induced proinflammation cytokines production in dendritic cells.^20,21^ Therefore, we designed the present study to explore the *in vivo* crosslink between cardiomyocytes and mTOR in the setting of sepsis and MSC therapy.

## Results

### Left ventricle function deteriorates in sepsis mice

**Figure 1:**
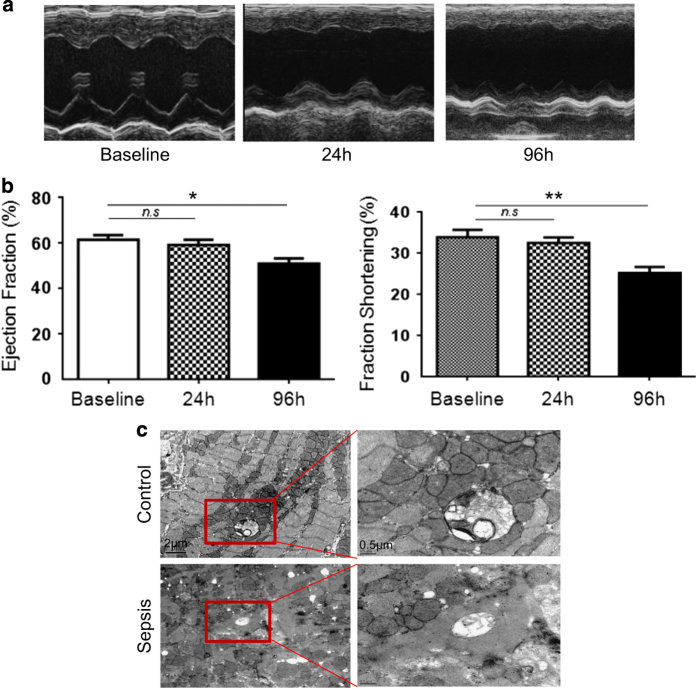
Sepsis causes murine’s LV function deteriorated and impairs the ultrastructure of the myocardium. (**a**) The LV function was measured by echocardiography after LPS intraperitoneal injection straightway, 24 and 96 h. Representative echocardiographic images were shown; (**b**) LV EF and LV FS after sepsis immediately as a baseline; 24 h post sepsis and 96 h post sepsis were measured. (**c**) Cardiomyocytes ultrastructure changes as evaluated by transmission electron microscopy. Scale bar, 2 and 0.5 *μ*m. The columns and error bars represent means and S.D. *n*=12 mice per group. *P<0.05 96 h post sepsis *versus* baseline, **P<0.05 96 h post sepsis *versus* baseline.

The level of serum inflammatory cytokines in sepsis group was noteworthy higher than sham group at post LPS injection 24 h. (IL-1*β*: 2276±208.6 *versus* 44.0±17.47, *n*=6；TNF-*α*: 2091±164.7 *versus* 31.00±13.45 *n*=6, *P*<0.05). The number of neutrophil and macrophage increased markedly in sepsis group as well. (neutrophil:1.57±0.40 ×10^9^ *versus* 6.22±0.65×10^9^ *n*=6; macrophage: 5.92±0.74×10^9^ *versus* 13.51±1.42×10^9^ *n*=6, *P*<0.05). However, there was no statistical significance of LV EF and LV FS between sepsis group and sham group in the initial 24 h after sepsis (Figure 1a and b). However, 96 h post LPS injection, the LPS-injected mice group had markedly decreases in LV EF (62.75±5.3 *versus* 52.28±3.4%, *P*<0.05) in comparison with baseline, as well as FS (33.45±2.7 *versus* 27.57±3.5, *P*<0.05) (Figure 1a and b). LVEDV (84.25±11.790% *versus* 66.10±9.105%, *P*<0.05) and LVESV (38.15±3.016 *versus* 26.13±3.402%, *P*<0.05) had significantly increased compared with baseline after 96 h of sepsis.

### Myocardium impairment and inflammation in mice sepsis model

**Figure 2:**
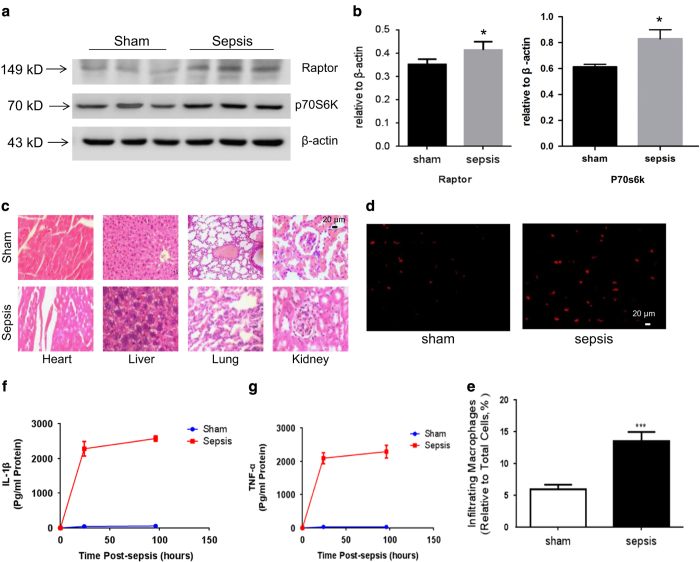
Sepsis causes murine`s myocardium impairment and inflammation. (**a**, **c**) The expression of Raptor was evaluated by western blot and normalized by expression levels of *β*-actin. (**b**) Hematoxylin and eosin stained heart, lung, liver and kidney histology from representative animals. Organs were fixed with 4% paraformaldehyde, embedded in paraffin, and cut into 5 mm-thick sections before being stained. Scale bar, 20 *μ*m (**d**) Immunofluorescence staining of macrophages in myocardium. Red fluorescence represent MAC-1^+^ macrophages. (**e**) The proportion of macrophages relative to total cells in 20 randomly selected fields (**f**) The expression of TNF-*α* and IL-1*β* in myocardium tissue were analyzed after sepsis by ELISA. The columns and error bars represent means and S.D. *n*=6 mice per group. ***P<0.001 *versus* sham.

Electron microscope was applied to study the ultrastructure of the myocardium. Electron microscope showed that the number of cardiomyocytes mitochondria was decreased as compared with the control group. Cristae disorientation and breakage were found in most swelling mitochondria (Figure 1c). The related protein expression was evaluated by western blot, and showed that the expression of Raptor and p70S6K in the sepsis group was increased compared with the control group (Figures 2a and c).

Inflammatory cells infiltration, exudation and structural damage could be found in multiple organs (Figure 2b). Neutrophil (1.57±0.40% *versus* 4.65±0.76%, *n*=6, *P*<0.001) and macrophage (5.92±0.74% *versus* 13.51±1.42%, *n*=6, *P*<0.001) infiltration in the myocardium significantly increased in the sepsis group compared with the sham group (Figure 2d). Inflammatory cytokines such as TNF-*α* and IL-1*β* in the heart tissue and in the serum were evaluated post LPS injection by the ELISA assay. The level of TNF-*α* and IL-1*β* in sepsis group was markedly higher than the sham group in both 24 and 96 h post LPS injection (Figures 2e and f).

### Characterization of BM-MSCs^Fluc+/GFP+^

**Figure 3:**
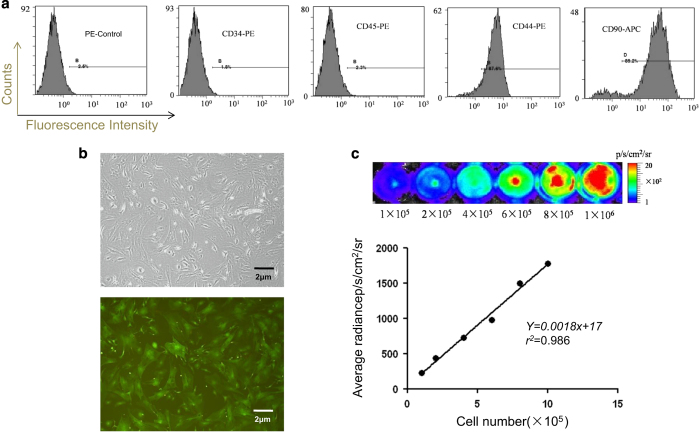
Characterization of BM-MSCs^Fluc+/GFP+^. (**a**) Flow cytometry results showed that BM-MSCs^Fluc+/GFP+^ were conformity with negative for CD34, CD45 and positive for CD44, CD90. (**b**) Fibroblast-like shaped BM-MSCs^Fluc+/GFP+^ were GFP-positive. Scale bar, 2 *μ*m (**c**) *Ex-vivo* BLI shows a linear relationship between cell number and Fluc reporter gene activity.

BM-MSCs^Fluc+/GFP+^ were isolated from Fluc-eGFP transgenic mice [Tg(Fluc-egfp)] and cultured *in vitro*, which expressed Fluc and GFP consistently within the cytosol. To evaluated the characteristics of BM-MSCs^Fluc+/GFP+^, cell surface markers were detected by flow cytometry. The data revealed that BM-MSCs^Fluc+/GFP+^ had higher expressions of MSC markers CD90, CD44 while lower expression of the hematopoietic progenitor cell marker CD34 and the leukocyte marker CD45 (Figure 3a). The expression of cluster differentiation of BM-MSCs^Fluc+/GFP+^ was consistent with that of MSCs.

BM-MSCs^Fluc+/GFP+^ exhibited a distinctive fibroblast-like morphology (Figure 3b). Fluorescence microscopy showed that the BM-MSCs^Fluc+/GFP+^ had bright eGFP fluorescence (Figure 3b). Adipogenic differentiation was assessed by Oil red O staining. As demonstrated by this method, generally positive adipocyte phenotype can be observed. Mineralized matrix deposition were stained by alizarin red S, which showed that the BM-MSCs^Fluc+/GFP+^ were able to differentiate into osteogenic cells.^22^ Bioluminescence imaging (BLI) quantification demonstrated a linear correlation between the number of BM-MSCs^Fluc+/GFP+^ and Fluc average radiance (Figure 3c) *in vitro*. These data suggested that BLI of Fluc was a reliable tool to quantitatively monitor the viability of the BM-MSCs^Fluc+/GFP+^ *in vivo*.

### Molecular Imaging and therapeutic effects of the engrafted BM-MSCs^Fluc+/GFP+^

**Figure 4:**
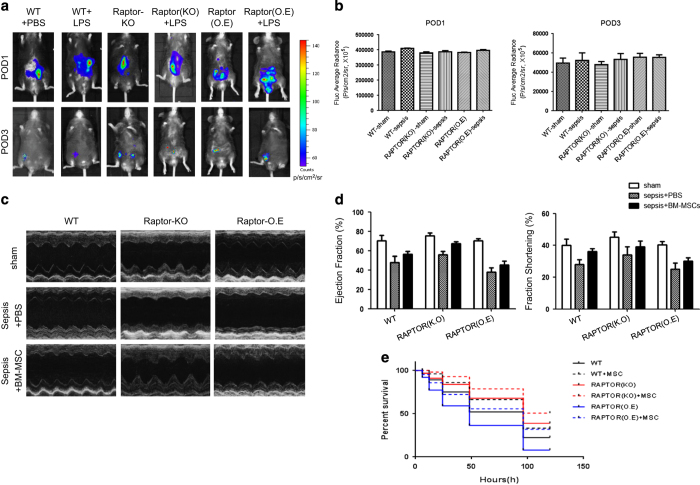
Molecular Imaging and therapeutic effects of the engrafted BM-MSCs^Fluc+/GFP+^. (**a**) The survival BM-MSCs^Fluc+/GFP+^ at 24 and 96 h post sepsis were monitoring by BLI (*n*=12 for each group). (**b**) The fluc average radiance was analyzed by BLI at 24 and 96 h post sepsis. (**c**, **d**) LV EF and LV FS at 96 h post sepsis were measured by echocardiography. Representative echocardiographic images at 96 h post sepsis were shown. (**e**) The survival proportion of representative groups after sepsis at indicated time points *P<0.05 *versus* WT group.

Sepsis mice were inoculated intraperitoneally with BM-MSCs^Fluc+/GFP+^ (2×10^6^ in 50 ul). Noninvasive BLI was applied to monitor the survival and proliferation of BM-MSCs^Fluc+/GFP+^ within 24 and 72 h post transplantation (Figure 4a). In the initial 24 h after the cells’ transplantation, the BLI showed that the signal was approximate 4×10^5^ p/s/cm^2^/sr and maintained stably, revealed no statistical significance among groups (data no shown). After 72 h, transplanted BM-MSCs^Fluc+/GFP+^ in all groups suffered a progressive death, the BLI signal of cell survival proportions in WT group compared with Raptor KO group (23.6±2.34% *versus* 28.3±5.46%, *P*>0.05) and Raptor overexpression group (23.6%±2.34 *versus* 24.8±3.01%, *P*>0.05) showed no statistical significance, and the BLI signal among all groups decayed to below 1×10^5^ p/s/cm^2^/sr (Figure 4b).

Echocardiography analyzed left ventricular (LV) ejection fraction (EF) and fraction shortening (FS) in all groups. We observed that the significant reduction of EF has occurred in all genotypes after LPS injection, while the Raptor KO-sepsis group showed less decrease of cardiac functional parameters (EF) compared with WT-sepsis group (EF: −17.86±4.09% *versus* −28.41±7.62%, *P*<0.05). The diastolic function was also significantly undermined in the three groups (WT-sepsis, Raptor KO-sepsis and Raptor O.E-sepsis) after LPS injection. However, the cardiac diastolic function in Raptor KO group was better preserved compared with the WT group (δFS: −8.1±1.36% *versus* −12.37±1.05, *P*<0.05) as well as compared with the Raptor overexpression group (δFS: −8.1±1.36% *versus* −11.82±1.79%, *P*<0.05; Figure 4e). After 96 h of cell-based treatment, echocardiography showed significant improvement in cardiac function in the WT group (EF:42.2±3.7% *versus* 59.2±3.5%, *P*<0.05), the Raptor KO group (EF:53.1±4.3 *versus* 60.9±1.5%) and the Raptor overexpression group (EF:40.6±2.0 *versus* 49.6±2.1%) respectively, while the Raptor KO- BMSC group had the optimum therapeutic effect (EF:60.9±1.5%). FS data showed that the diastolic function of the WT group (29.3±1.7% *versus* 35.9±3.1%) and the Raptor KO group (33.7±4.3 *versus* 38.9±2.8%) with sepsis was slightly improved after BM-MSC treatment (*P*<0.05), while the Raptor overexpression group (28.1±2.6 *versus* 23.1±2.5%) showed no obvious amelioration treated with BM-MSC (*P*>0.05; Figures 4c and d). After 96 h of cell-based treatment, echocardiography showed significant improvement in cardiac function in the WT group (EF:42.2±3.7% *versus* 59.2±3.5%, *P*<0.05), the Raptor KO group (EF:53.1±4.3 *versus* 60.9±1.5%) and the Raptor overexpression group (EF:40.6±2.0 *versus* 49.6±2.1%) respectively, while the Raptor KO- BMSC group had the optimum therapeutic effect (EF:60.9±1.5%). FS data showed that the diastolic function of the WT group (29.3±1.7% *versus* 35.9±3.1%) and the Raptor KO group (33.7±4.3 *versus* 38.9±2.8%) with sepsis was slightly improved after BM-MSC treatment (*P*<0.05), while the Raptor overexpression group (28.1±2.6 *versus* 23.1±2.5%) showed no obvious amelioration treated with BM-MSC (*P*>0.05; Figures 4c and d)

Intriguingly, the therapeutic effects of the engrafted BM-MSCs^Fluc+/GFP+^ showed a significant increase in survival rate in the cardiac-conditional ablation of Raptor (component of mTORC1 complex; Raptor KO) mice. Kaplan–Meyer curve of survival rate showed that the mice’s survival proportion of the Raptor KO group was significantly higher than the WT group in both 24 and 96 h (89.23 *versus* 75.30%, 73.15 *versus* 60.07%, *P*<0.05, *n*=40) after cell-based treatment. However, the proportion of Raptor overexpression group was lower than the WT and the Raptor KO group (55 *versus* 75%, 60 *versus* 75%, *P*<0.05, *n*=40; Figure 4f).

**Figure 5:**
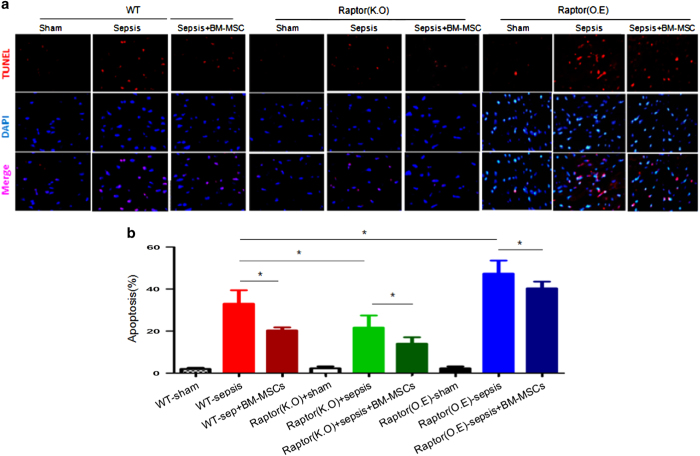
Anti-apoptotic effect of MSCs on cardiomyocytes after sepsis. (**a**) TUNEL was administrated by the apoptosis assay kit, and positive myocytes were indicated in red and nucleis in blue. (**b**) Quantitative analysis of apoptotic cardiomyocytes isolated from cardiac tissue in sepsis and each AI was assessed in 20 randomly selected fields. *P<0.05.

Cardiomyocytes apoptosis in sepsis were evaluated by TUNEL. The apoptosis index (AI), as assessed by TUNLE assay, was significantly lower in the Raptor KO-sepsis+BM-MSCs group compared with the WT-sepsis+BM-MSCs group (*P*<0.05) and the WT-sepsis+Raptor overexpression group (*P*<0.05; Figures 5a and b). Raptor KO-sepsis group had also significantly decreased the AI in comparison with WT-sepsis group. Conversely, Raptor overexpression-sepsis group markedly increased the AI. Meanwhile, MSCs efficacy in Raptor overexpression group had been abolished compared with WT-sepsis+ BM-MSC group.

### mTORC1 and mTORC2 are both involved in the immunomodulatory function of BM-MSCs

**Figure 6:**
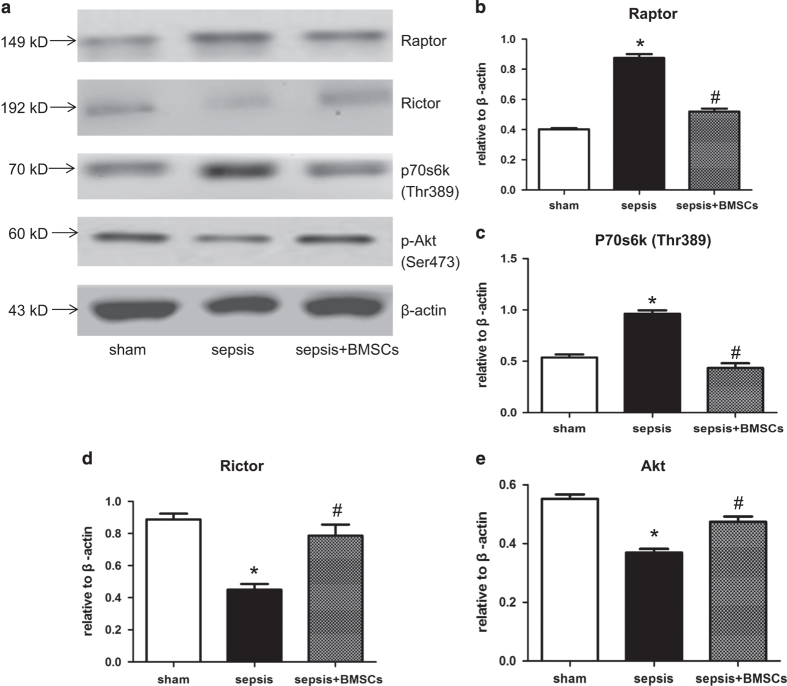
BMSCs-activated mTORC1/mTORC2 signal pathways. (**a**) Western blot was performed with the protein lysates and mTOR. (**b**)Immunoprecipitates prepared from the lysates derived from LV tissues on POD3 for mTORC1/mTORC2 pathways analysis. Figures are representative immunoblots of at least five different samples. (**a**) Protein expression was quantified by the integrated optical density (IOD) ratio of: (**b**) Raptor; (**c**) Rictor; (**d**) S6K(Thr389); and (**e**) Akt. Error bars represent mean±S.D. *P<0.05 *versus* Sham; ^#^*P*<0.05 *versus* sham; ^#^*P*<0.05 *versus* sepsis. BMSCs, bone marrow-derived mesenchymal stromal cell; mTORC, complex of mammalian target of rapamycin; PBS, phosphate-buffered saline; S6K (p70S6K), ribosomal protein S6 kinases.

Immunoprecipitates prepared from the lysates derived from LV tissues on POD3 for mTORC1/mTORC2 pathways analysis. Western blot showed that the expression of Raptor and p70S6k in myocardium were upregulated in the sepsis group, while the level of Rictor and p-Akt were downregulated. Nevertheless, administration of BM-MSCs intraperitoneally downregulated Raptor and p70S6k compared with the sepsis group, whereas the Rictor and p-Akt were relatively upregulated (Figure 6).

### BM-MSCs’ anti-inflammation function is correlated with mTORC1

**Figure 7:**
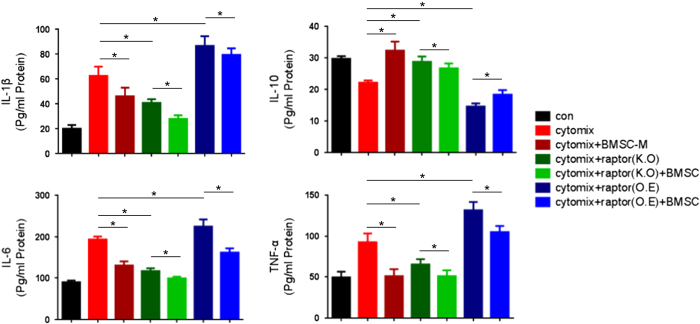
mTORC1 is involved in the immunomodulatory function of BM-MSC. The expression of IL-1*β*, IL-6, TNF-*α* and IL-10 in culture medium in respective group was evaluated by ELISA. *P<0.05.

To further explore the effect of the immunomodulatory effect of BM-MSC, we designed the *in vitro* experiment. Cytomix secreted by LPS-stimulated macrophages were added to H9c2 cells (in Supplementary Materials)， co-cultured with/without BM-MSCs. Analysis of inflammatory cytokines revealed that BM-MSC’s anti-inflammation function is correlated with mTORC1. ELISA was used to measure IL-1*β*, TNF-*α*, IL-6 and IL-10 expression *in vitro*. The group downregulated the expression of IL-1*β* and TNF-*α*, which are known proinflammatory cytokines that induce acute cell death,^20,23^ and upregulated the expression of IL-10, which are known anti-inflammatory cytokine that enhanced the cell survival *in vivo*.^24^ The anti-inflammation function had been enhanced by Raptor KO simultaneously (Figure 7). Furthermore, although BM-MSCs sacrificed, Raptor KO still retained the anti-inflammatory efficacy through modulating the expression of cytokines. However, Raptor overexpression inverted the containing of cytokines in cardiomyocytes, which was consistent with the result of TUNEL.

### BM-MSCs modulates inflammatory response through mTORC1-p70S6K/ mTORC2-Akt signal pathway.

To further explore the mechanism of the immunomodulatory effect of BM-MSC and specify the relationship among engrafted BM-MSCs, macrophage and cardiomyocytes in sepsis, we designed the *in vitro* experiment. Cytomix secreted by LPS-stimulated macrophages were added to H9c2 cells(in Supplementary Materials), co-cultured with/without BM-MSCs. We examined the mTORC1-p70S6K and mTORC2-Akt signal pathways using western blotting. Cytomix administration increased the level of Toll-like receptor 4 (TLR-4) and the downstream NF-*κ*B, and these effects were attenuated by addition of BM-MSCs. Meanwhile, the expression of Raptor and p70S6k were upregulated, accompanied with the addition of Cytomix, and the level of Rictor and *p*-Akt were downregulated. The apoptosis associated protein caspase-3 was also increased after cytomix addition. MSC altered the level of Raptor, p70S6k, Rictor, *p*-Akt and caspase-3.

**Figure 8:**
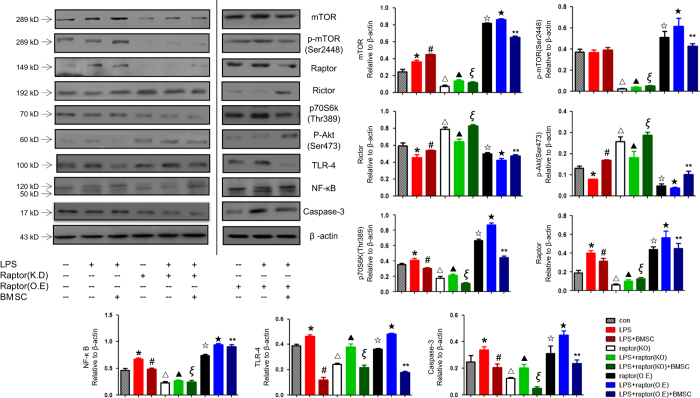
BM-MSC modulates inflammation response through mTORC1-p70S6K/mTORC2-Akt signal pathway. The expression of mTOR, *p*-mTOR, Raptor, Rictor, *p*-Akt, p70S6K, NF-*κ*B, TLR-4 and Caspase-3 in H9c2 cell lysate were measured by western blot and normalized by expression levels of *β*-actin. *P<0.05 *versus* control group, ^#^*P*<0.05 *versus* control group, ^▵^*P*<0.05 *versus* control group, ^▲^*P*<0.05 *versus* control group, *^ξ^P*<0.05 *versus* control group, ^☆^*P*<0.05 *versus* control group, ^★^*P*<0.05 *versus* control group, **P<0.05 *versus* control group.

Raptor KO decreased the expression of TLR-4 and NF-*κ*B, as well as Raptor, p70S6k and caspase-3, and these effects were reinforced as the combination of BM-MSC and Raptor KO. However, the effects of BM-MSC were abolished as the Raptor overexpression (Figure 8).

## Discussion

Mortality of sepsis has declined in the past decade, most likely due to systematic advanced management of severe sepsis, and an improved understanding of sepsis mechanism. Yet, the disease remains a significant clinical problem among surgical and trauma patients.^2,25^ Cellular biology treatment, especially MSC-based therapy was considered as a promising approach for tissue regeneration, immunomodulatory, and sepsis treatment recent years. In the present study, we found that BM-MSC could modulate the host inflammatory response in sepsis-induced cardiomyocytes by exerting an anti-apoptotic effect and ameliorating cardiac function through the mTORC1-p70S6K pathway.

Recent evidence suggests that MSCs may exhibit immunosuppressive or immunomodulatory properties, and can be easily obtained and isolated from bone marrow.^26–28^ Furthermore, the low immunogenicity and immunoprivileged properties of MSCs may allow allogeneic or even xenogeneical treatment.^29,30^ This raises the possibility for non-autologous transplantation of MSCs as a therapeutic strategy. We previously explored the potential treatment mechanism of MSC by monitoring the fate of transplanted BM-MSC using molecular imaging strategy, which provided valuable insight into the *in vivo* kinetics of engrafted cells.^17,31^ BLI is an accurate, sensitive, approach for noninvasive stem cell tracking. Relying on BLI, we visualized the fates of BM-MSC following intraperitoneal inoculation. It showed no statistical significance of BM-MSCs survival among the different groups. We also isolated heart, liver, lung and kidney and investigated in the BLI system at 24 and 72 h post intraperitoneal inoculation. Amazingly, little BM-MSCs was found in these major organs. However, the therapeutic effect still remained. The cardiac function in Raptor KO and the Raptor KO+BM-MSC groups were both well preserved in the setting of sepsis. What’s more, the therapeutic effect was abolished by the overexpression of Raptor, which strongly indicates that mTORC1 is a pivotal checkpoint in MSC anti-inflammation therapy.

Previous investigations have demonstrated that MSC treatment reduced the levels of proinflammatory cytokines (i.e., IFN-γ, TNF-*α*, IL-1*β* or IL-6) in several organs including serum, liver, lung, intestine and myocardium,^32–34^ Although related to different experimental settings as a result of a conflicting expression of IL-10, there were evidences that the increasing level of IL-10 has an important role in the therapeutic effects of MSCs in sepsis.^23,35,36^ Interestingly, recent investigations have also demonstrated the expression of TLRs in MSCs and that these receptors may play a role in mediating many of the beneficial actions of stem cells, which lends further credence to the theory that stem cells may offer benefit in the treatment of sepsis. In our experiment, the engrafted MSCs reduced the expression of TLR-4 in cardiomyocytes, which has been verified in immune cells and MSCs, but not in cardiomyocytes. It is suggested that MSCs may conduct a ‘neutralization’ effect, which means that the LPS integrates to TLR-4 on the surface of MSC instead of cardiomyocytes, alleviating the detrimental effects of LPS to cardiomyocytes. Furthermore, NF-*κ*B, the initiated transcription of genes associated with inflammation, also declined as the downregulation of TLR-4.

mTOR has a pivotal role in cells survival and inflammation,^18^ while mTORC1/mTORC2, 2 distinct multiprotein complexes, may play different roles in cellular responsiveness.^18^ Our data demonstrated that mTORC1/mTORC2 was responsible for mediating BM-MSC-directed inflammatory modulation and anti-apoptosis effects in sepsis. Our results demonstrated that MSC modulated the level of proinflammatory cytokines, which increased in the Raptor overexpression group and decreased in the Raptor KO group. Meanwhile, the anti-inflammatory cytokine IL-10 also decreased in Raptor overexpression group. Engrafted BM-MSCs promoted the activation of mTORC1/mTORC2 and their respective downstream signaling pathways, namely, p70S6K and Akt. mTORC1-p70S6K axis exhibits a proinflammatory property, as mTORC1-p70S6K activation by endotoxin contributes to cytokine upregulation as a consequence of lethal inflammation. In addition, we also found the upregulation of Rictor, the functional component of mTORC2, leads to the activation of Akt, which could modulate cellular activation, inflammatory response and apoptosis, and downregulates the downstream apoptosis-related protein caspase-3. Intriguingly, a delicate balance of mTORC1/mTORC2 may exist, as the total activation of mTOR is stable along with the up and down regulation of Raptor/Rictor.

Although our study shows a promising therapy with potential clinical relevance, several limitations exist. First, there are several sepsis models such as toxemia models, bacterial infection models and host barrier disruption models; however, there is no single one that can fully mimic the course of human disease as the timing of disease progression and lacking of supportive therapeutic intervention, in particular in small animal models. Second, BLI can be applied as a powerful tool for *in vivo* tracking of stem cells; however, the limitation for imaging of deep tissues *in vivo* challenges its future clinical application. Third, clinical application of MSCs is confronted with the insufficient supplement of source; therefore, a specialized autologous stem cell bank for individualized treatment needs to be available.

**Figure 9:**
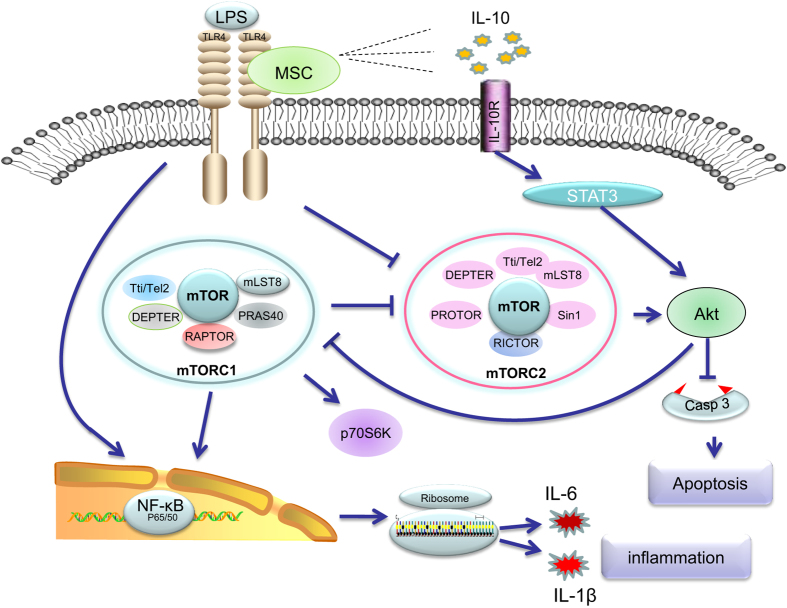
BM-MSC modulates inflammation response through mTORC1-p70S6K/mTORC2-Akt Signal Pathway. MSC modulated the level of proinflammatory cytokines. Meanwhile, the anti-inflammatory cytokine IL-10 also decreased in Raptor overexpression group. Engrafted BM-MSCs promoted the activation of mTORC1/mTORC2 and their respective downstream signaling pathways, namely, p70S6K and Akt. mTORC1-p70S6K axis exhibits a proinflammatory property, as mTORC1-p70S6K activation by endotoxin contributes to cytokine upregulation as a consequence of lethal inflammation.^42,43^ The functional component of mTORC2, leads to the activation of Akt, which could modulate cellular activation, inflammatory response, and apoptosis,^44,45^ and down regulates the downstream apoptosis-related protein caspase-3. Intriguingly, a delicate balance of mTORC1/mTORC2 may exist, as the total activation of mTOR is stable along with the up- and downregulation of Raptor/Rictor.

In conclusion, our studies demonstrated that BM-MSC can modulate sepsis-induced inflammation through mTORC1-S6K and mTORC2-Akt pathway, alleviating sepsis-induced cardiac dysfunction. Furthermore, a subtle balance of mTORC1 and mTORC2 was revealed, which was indispensable for the modulating of inflammation. In addition, TLR-4/NF-*κ*B pathway was also involved in this process, and BM-MSC could decrease this proinflammatory signal pathway (Figure 9). These data leads us to better understand the mechanism of MSC’s immunomodulatory effect, facilitating the progress of clinical translation of cell-based sepsis treatment.

### Innovation

MSC-based treatment was focused on the sepsis treatment for past few years, yet the defined mechanism of its effect was poorly elucidated. *In vivo* BLI can be applied as a noninvasive tool for monitoring the fate of transplantation stem cells. Furthermore, we demonstrated for the first time that the mTORC1-p70S6K and mTORC2-Akt pathways may be involved in the MSC’s immunomodulatory effect, which leads to the suppression of proinflammatory cytokines, and promotion of anti-inflammatory cytokine. Therefore, our findings may facilitate the further application of stem cell for the biological treatment of sepsis.

## Materials and Methods

### Animals

The source of firefly luciferase and enhanced green fluorescent protein-positive transgenic mice [Tg(Fluc-eGFP)] was consist with our previous study.^10^ The mice were bred on a C57BL/6a background to constitutively expressing both firefly luciferase (Fluc) and enhanced green fluorescence protein (eGFP) in all tissues and organs. Eight week-old wild-type mice C57BL/6a background (WT), conditional deletion of Raptor in cardiomyocytes (Raptor KO) and were used to construct sepsis model. *RPTOR* ^floxed/floxed^ (Jackson Laboratory, Bar Harbor, ME, USA Stock Number: 013188) were mated with B6.FVB-Tg Myh6-cre (Jackson Laboratory, Stock Number: 011038), and crossbred for more than three generations to make the Raptor^−/−^ (Raptor KO) mice in the offspring. These Raptor^−/−^ (Raptor KO)mice have been backcrossed with wild-type C57BL/6a mice for nine or more generations, ensuring >99% of C57BL/6a genetic background. Raptor overexpressed mice were established by cardiac-specific injection of adenovirus(specified in Supplementary Material). The use of laboratory animals and all procedures were performed in accordance with the National Institutes of Health Guidelines on the Use of Laboratory Animal. Experimental protocols and animal care methods were approved by the Fourth Military Medical University Committee on Animal Care.

### Isolation, culture and identification of BM-MSCs^Fluc+/GFP+^

Bone marrow-derived MSCs, BM-MSCs^Fluc+/GFP+^, were isolated from Tg[Fluc-egfp] mice with a modified procedure as described previously and cultured *in vitro*.^37^ In brief, dissecting femurs, humeral and tibial bones from surrounding muscle and connective tissue, the bone marrow was eluted with a high-glucose Dulbecco`s modified Eagle’s medium (DMEM, Hyclone, Logan, UT, USA), 10% fetal bovine serum (FBS, Gibco, Australia Origin), 1% penicillin/streptomycin and harvested. Stromal cells separated from precipitation after centrifugation. The cells were resuspended in DMEM/F12 (1 : 1; Hyclone) supplemented with 15% FBS and basic fibroblast growth factor (bFGF, Sigma, Billerica, MA, USA). After 48 h motionless at 37 °C and 5% CO_2_, non-adherent cells were swept off by phosphate-buffered saline (PBS). Culture media was changed per 48 h. Exponential phase cells were used for further experiments.

Immunophenotype and multi-potency were identified by flow cytometry and chemical induction, respectively. Stem cell markers CD44, CD90, CD34, CD31 and CD45 were assessed by flow cytometry. Adipogenic differentiation was induced by adipogenic media (*α*MEM with 10% FCS, 1% antibiotics, 50 *μ*M indomethacin, 0.5 mM IBMX and 1 *μ*M dexamethasone, Sigma), followed by oil red O staining after 21 days incubating. Osteogenesis differentiation of BM-MSC was induced in osteogenic medium (10% FBS, 0.1 *μ*M dexamethasone, 10 mM b-glycerophosphate and 0.2 mM ascorbic acid in *α*MEM), and the degree of extracellular matrix calcification was estimated by an alizarin red S stain after 21 days.

### Adenoviral construction and infection

The adenovirus was purchased from Neuron Biotech co. Ltd., Shanghai, China. Recombinant adenoviruses were generated with HEK293 cell Raptor gene cDNAs sub-cloned into the pShuttle-cytomegalovirus vector using the AdEasy XL Adenoviral Vector System (Santa Clara, CA, USA). H9C2 cells were infected with adenoviruses, as described previously.^38^

### Cell lines and culture

H9C2 cells were maintained in high-glucose DMEM (Hyclone) and containing 10% FBS, 1% penicillin/streptomycin, at 37 °C and 5% CO_2_. Culture media was changed per 48 h, and exponential phase cells were used for experiments.

BM-MSCs and H9C2 cells were co-cultured in a Transwell system. When H9C2 cells were grown in a tight monolayer at 96–120 h, the cells were washed and serum starved with plain medium for 24 h. To determine whether mTORC1 is involved in the inflammatory process, we knocked down or overexpressed the Raptor in H9C2 with mTORC1 siRNA or adenovirus infection, respectively. BM-MSCs were then plated in the bottom compartment of the Transwell at a density of 2.5×10^5^ cells/well with indirect contaction with H9C2 cells. The H9C2 cells and BM-MSCs were exposed to a mixture of three different cytokines referred to as cytomix (IL-1*β*, TNF-*α* and IFN-g, 50ng/ml each; R&D Systems, Minneapolis, MN, USA). The cytomix was added simultaneously, and conditions were maintained for 24 h.

### *In vivo* BLI for monitoring of engrafted BM-MSCs^Fluc+/GFP+^

*In vivo* BLI was applied to monitor the survival and proliferation of transplanted BM-MSCs^Fluc+/GFP+^. Bioluminescence signal was detected by Xenogen *in Vivo* Imaging System (Caliper Life Sciences, USA). Mice were intraperitoneally injected with D-luciferin (150 mg/kg) and anesthetized with 2% isoflurane in the imaging chamber. Baseline images were acquired at the initial, thereafter, mice were imaged for 10 min with 1 min acquisition intervals until the peak signal was captured at post LPS injection 24, 48, 72 and 96 h. Bioluminescent signals were analyzed by Living Image software and were quantified as average radiance in photons/s/cm^2^/sr.

### Sepsis model and evaluation of cardiac function

A single injection of high-dose LPS (20 mg/kg) was administrated to subject mice. After 24 h, mice with an increase of white blood cell counts of >12×10^9^ or <4×10^9^, plasma C-reactive protein over 2 S.D. and IL-1*β*＞1500 pmol/l were selected as sepsis model. Echocardiography was performed after LPS intraperitoneal injection as a baseline, and then, post LPS injection 24 and 96 h were also measured. Mice were anesthetized with a mixture of 2% isoflurane and oxygen, measuring by an echocardiography system. The LV function parameters were measured and using of computer algorithms to calculate LV ejection fraction (EF) and fractional shortening (FS). All data represent the mean of five consecutive cardiac cycles.

### Apoptosis assessment

Myocardium was harvested and apoptosis was determined by terminal deoxynu-cleotidyl transferase-mediated dUTP-biotin nick end labeling (TUNEL) staining, using a TUNEL Apoptosis Assay Kit. (R&D, Switzerland) TUNEL staining was performed with fluoresce in-dUTP for apoptotic cell nuclei and 4`6-diamidino-2-phenylindole (DAPI) to stain all cell nuclei. Apoptosis Index (AI), the number of TUNEL-positive cells divided by the total cells per field, was examined. Each AI was assessed in 20 randomly selected fields.

### Assessment of protein expression and cytokines

Protein samples from the H9C2 cells extracts were separated by 12% SDS-PAGE and transferred to nitrocellulose (NC) membranes. The membrane was blocked by Tris-buffered saline Tween-20 (TBST) containing 5% milk for 1 h at room temperature. Then, immunoblotting was performed with appropriate primary antibodies at 4 °C overnight. Primary antibodies used in this study were: phosphor-mTOR (Ser2448) (rabbit polyclonal IgG, Cell Signaling Technology, Danvers, MA, USA, 1 : 1000); anti-mTOR (rabbit polyclonal IgG, Cell Signaling Technology, 1 : 1000); *p*-Akt (Ser473; rabbit polyclonal IgG, Cell Signaling Technology, 1 : 1000); anti-Rictor (rabbit monoclonal IgG, Cell Signaling Technology, 1 : 500); anti-Raptor (rabbit monoclonal IgG, Cell Signaling Technology, 1 : 500); anti-TLR-4 (rabbit polyclonal IgG, Cell Signaling Technology, 1 : 500); anti-caspase-3 (rabbit monoclonal IgG, abcam, Cambridge, MA, USA, 1 : 500); anti-NF-*κ*B (rabbit polyclonal IgG, Santa Cruze Biotechnology, Dallas, TX, USA, 1 : 200); anti-p70S6K (rabbit polyclonal IgG, Cell Signaling Technology Biotechnology, 1 : 500); and anti *β*-actin (rabbit monoclonal IgG, Santa Cruze, 1 : 1500). After washing three times with TBST for 5 min, the blot further incubated with appropriate secondary antibody conjugated with horseradish peroxidase, respectively at room temperature for 60 min, bands were visualized using an enhanced chemiluminescence system. Densitometric analysis of western blots was carried out using Vision Works software (version 6.7.1, Upland, CA, USA).

TNF-*α*, IL-1*β*, IL-6 and IL-10 levels in myocardial tissue and cell culture supernatants were determined by enzyme-linked immunosorbent assay (ELISA) using commercially available kits (Sigma). ELISA was performed according to the manufacturer’s instructions.

### Immunofluorescence staining of macrophages

Histological sections of myocardium was harvested and washed three times with PBS, blocked with blocking buffer (1% BSA and 0.3% TritonX-100 in PBS) for 30 min and then incubated with the goat polyclonal anti-mouse MAC-3 antibody (Biolegend Co., San Diego, CA, USA, 1 : 100 dilution in blocking buffer) for 1 h at 37 °C. After extensive washing, the histological sections were incubated for 45 min with Cy3-conjugated anti-goat IgG secondary antibody(1 : 50 dilution in blocking buffer) at 37 °C. After washing, the slides were placed in mounting media. As a control, tissue slides were stained with secondary antibody alone. The experiment also settled negative control. The tissue slides were then examined using a fluorescence microscope.

### Statistical analysis

The results are expressed as mean±S.D. Prism 5.0 (GraphPad Software Inc, La Jolla, CA, USA) was used to perform the one-way analysis of variance for the comparisons of parameters among three or more groups, or the Student’s *t*-test between two groups. Linear regression analysis was performed to determine the correlation between two variables; *P*-values<0.05 were considered statistically significant.

## References

[bib1] Dellinger RP, Levy MM, Rhodes A, Annane D, Gerlach H, Opal SM et al. Surviving Sepsis Campaign Guidelines Committee including The Pediatric, S. Surviving Sepsis Campaign: international guidelines for management of severe sepsis and septic shock, 2012. Intensive Care Med 2013; 39: 165–228. 23361625 10.1007/s00134-012-2769-8 PMC7095153

[bib2] American College of Chest Physicians/Society of Critical Care Medicine Consensus Conference: definitions for sepsis and organ failure and guidelines for the use of innovative therapies in sepsis. Crit Care Med 1992; 20: 864–874. 1597042

[bib3] Fan W, Cheng K, Qin X, Narsinh KH, Wang S, Hu S et al. mTORC1 and mTORC2 play different roles in the functional survival of transplanted adipose-derived stromal cells in hind limb ischemic mice via regulating inflammation *in vivo*. Stem Cells 2013; 31: 203–214. 23081858 10.1002/stem.1265

[bib4] Zhang Z, Li S, Cui M, Gao X, Sun D, Qin X et al. Rosuvastatin enhances the therapeutic efficacy of adipose-derived mesenchymal stem cells for myocardial infarction via PI3K/Akt and MEK/ERK pathways. Basic Res Cardiol 2013; 108: 333. 23386286 10.1007/s00395-013-0333-5

[bib5] Weil BR, Markel TA, Herrmann JL, Abarbanell AM, Kelly ML, Meldrum DR. Stem cells in sepsis. Ann Surg 2009; 250: 19–27. 19561461 10.1097/SLA.0b013e3181a77b9c

[bib6] Wang Y, Li C, Cheng K, Zhang R, Narsinh K, Li S et al. Activation of liver X receptor improves viability of adipose-derived mesenchymal stem cells to attenuate myocardial ischemia injury through TLR4/NF-kappaB and Keap-1/Nrf-2 signaling pathways. Antioxid Redox Signal 2014; 21: 2543–2557. 24915051 10.1089/ars.2013.5683 PMC4245883

[bib7] Rodriguez AM, Elabd C, Amri EZ, Ailhaud G, Dani C. The human adipose tissue is a source of multipotent stem cells. Biochimie 2005; 87: 125–128. 15733747 10.1016/j.biochi.2004.11.007

[bib8] Romanov YA, Svintsitskaya VA, Smirnov VN. Searching for alternative sources of postnatal human mesenchymal stem cells: candidate MSC-like cells from umbilical cord. Stem Cells 2003; 21: 105–110. 12529557 10.1634/stemcells.21-1-105

[bib9] Cao F, Lin S, Xie X, Ray P, Patel M, Zhang X et al. *In vivo* visualization of embryonic stem cell survival, proliferation, and migration after cardiac delivery. Circulation 2006; 113: 1005–1014. 16476845 10.1161/CIRCULATIONAHA.105.588954 PMC4701384

[bib10] Laplante M, Sabatini DM. mTOR signaling in growth control and disease. Cell 2012; 149: 274–293. 22500797 10.1016/j.cell.2012.03.017 PMC3331679

[bib11] Wullschleger S, Loewith R, Hall MN. TOR signaling in growth and metabolism. Cell 2006; 124: 471–484. 16469695 10.1016/j.cell.2006.01.016

[bib12] Ohtani M, Nagai S, Kondo S, Mizuno S, Nakamura K, Tanabe M et al. Mammalian target of rapamycin and glycogen synthase kinase 3 differentially regulate lipopolysaccharide-induced interleukin-12 production in dendritic cells. Blood 2008; 112: 635–643. 18492954 10.1182/blood-2008-02-137430 PMC2481549

[bib13] Muller-Ehmsen J, Krausgrill B, Burst V, Schenk K, Neisen UC, Fries JW et al. Effective engraftment but poor mid-term persistence of mononuclear and mesenchymal bone marrow cells in acute and chronic rat myocardial infarction. J Mol Cell Cardiol 2006; 41: 876–884. 16973174 10.1016/j.yjmcc.2006.07.023

[bib14] Loboda A, Stachurska A, Florczyk U, Rudnicka D, Jazwa A, Wegrzyn J et al. HIF-1 induction attenuates Nrf2-dependent IL-8 expression in human endothelial cells. Antioxid Redox Signal 2009; 11: 1501–1517. 19254160 10.1089/ars.2008.2211

[bib15] Mocellin S, Marincola FM, Young HA. Interleukin-10 and the immune response against cancer: a counterpoint. J Leukoc Biol 2005; 78: 1043–1051. 16204623 10.1189/jlb.0705358

[bib16] Mayr FB, Yende S, Angus DC. Epidemiology of severe sepsis. Virulence 2014; 5: 4–11. 24335434 10.4161/viru.27372 PMC3916382

[bib17] Angus DC, Linde-Zwirble WT, Lidicker J, Clermont G, Carcillo J, Pinsky MR. Epidemiology of severe sepsis in the United States: analysis of incidence, outcome, and associated costs of care. Crit Care Med 2001; 29: 1303–1310. 11445675 10.1097/00003246-200107000-00002

[bib18] Rahavi H, Hashemi SM, Soleimani M, Mohammadi J, Tajik N. Adipose tissue-derived mesenchymal stem cells exert *in vitro* immunomodulatory and beta cell protective functions in streptozotocin-induced diabetic mice model. J Diabetes Res 2015; 2015: 878535. 25893202 10.1155/2015/878535 PMC4393922

[bib19] Horwitz EM, Gordon PL, Koo WK, Marx JC, Neel MD, McNall RY et al. Isolated allogeneic bone marrow-derived mesenchymal cells engraft and stimulate growth in children with osteogenesis imperfecta: Implications for cell therapy of bone. Proc Natl Acad Sci USA 2002; 99: 8932–8937. 12084934 10.1073/pnas.132252399 PMC124401

[bib20] Berlier JL, Rigutto S, Dalla Valle A, Lechanteur J, Soyfoo MS, Gangji V et al. Adenosine triphosphate prevents serum deprivation-induced apoptosis in human mesenchymal stem cells via activation of the MAPK signaling pathways. Stem Cells 2015; 33: 211–218. 25183652 10.1002/stem.1831

[bib21] Guo CH, Han LX, Wan MR, Deng GJ, Gan JH. Immunomodulatory effect of bone marrow mesenchymal stem cells on T lymphocytes in patients with decompensated liver cirrhosis. Genet Mol Res 2015; 14: 7039–7046. 26125913 10.4238/2015.June.26.13

[bib22] Agrawal H, Shang H, Sattah AP, Yang N, Peirce SM, Katz AJ. Human adipose-derived stromal/stem cells demonstrate short-lived persistence after implantation in both an immunocompetent and an immunocompromised murine model. Stem Cell Res Ther 2014; 5: 142. 25523792 10.1186/scrt532 PMC4445497

[bib23] Fruman DA, Cantley LC. Phosphoinositide 3-kinase in immunological systems. Semin Immunol 2002; 14: 7–18. 11884226 10.1006/smim.2001.0337

[bib24] Cao F, Li Z, Lee A, Liu Z, Chen K, Wang H et al. Noninvasive *de novo* imaging of human embryonic stem cell-derived teratoma formation. Cancer Res 2009; 69: 2709–2713. 19318556 10.1158/0008-5472.CAN-08-4122 PMC2866177

[bib25] Zhao X, Liu D, Gong W, Zhao G, Liu L, Yang L et al. The toll-like receptor 3 ligand, poly(I:C), improves immunosuppressive function and therapeutic effect of mesenchymal stem cells on sepsis via inhibiting MiR-143. Stem Cells 2014; 32: 521–533. 24105952 10.1002/stem.1543

[bib26] Krasnodembskaya A, Samarani G, Song Y, Zhuo H, Su X, Lee JW et al. Human mesenchymal stem cells reduce mortality and bacteremia in gram-negative sepsis in mice in part by enhancing the phagocytic activity of blood monocytes. Am J Physiol Lung Cell Mol Physiol 2012; 302: L1003–L1013. 22427530 10.1152/ajplung.00180.2011 PMC3362255

[bib27] Mei SH, Haitsma JJ, Dos Santos CC, Deng Y, Lai PF, Slutsky AS et al. Mesenchymal stem cells reduce inflammation while enhancing bacterial clearance and improving survival in sepsis. Am J Respir Crit Care Med 2010; 182: 1047–1057. 20558630 10.1164/rccm.201001-0010OC

[bib28] Sepulveda JC, Tome M, Fernandez ME, Delgado M, Campisi J, Bernad A et al. Cell senescence abrogates the therapeutic potential of human mesenchymal stem cells in the lethal endotoxemia model. Stem Cells 2014; 32: 1865–1877. 24496748 10.1002/stem.1654 PMC4209016

[bib29] Pevsner-Fischer M, Morad V, Cohen-Sfady M, Rousso-Noori L, Zanin-Zhorov A, Cohen S et al. Toll-like receptors and their ligands control mesenchymal stem cell functions. Blood 2007; 109: 1422–1432. 17038530 10.1182/blood-2006-06-028704

[bib30] Shi L, Wang JS, Liu XM, Hu XY, Fang Q. Upregulated functional expression of Toll like receptor 4 in mesenchymal stem cells induced by lipopolysaccharide. Chin Med J (Engl) 2007; 120: 1685–1688. 17935670

[bib31] Delgoffe GM, Pollizzi KN, Waickman AT, Heikamp E, Meyers DJ, Horton MR et al. The kinase mTOR regulates the differentiation of helper T cells through the selective activation of signaling by mTORC1 and mTORC2. Nat Immunol 2011; 12: 295–303. 21358638 10.1038/ni.2005 PMC3077821

[bib32] Di R, Wu X, Chang Z, Zhao X, Feng Q, Lu S et al. S6K inhibition renders cardiac protection against myocardial infarction through PDK1 phosphorylation of Akt. Biochem J 2012; 441: 199–207. 21906027 10.1042/BJ20110033

[bib33] Lorne E, Zhao X, Zmijewski JW, Liu G, Park YJ, Tsuruta Y et al. Participation of mammalian target of rapamycin complex 1 in Toll-like receptor 2- and 4-induced neutrophil activation and acute lung injury. Am J Respir Cell Mol Biol 2009; 41: 237–245. 19131641 10.1165/rcmb.2008-0290OC PMC2715911

[bib34] Franke TF, Kaplan DR, Cantley LC. PI3K: downstream AKTion blocks apoptosis. Cell 1997; 88: 435–437. 9038334 10.1016/s0092-8674(00)81883-8

[bib35] Buras JA, Holzmann B, Sitkovsky M. Animal models of sepsis: setting the stage. Nat Rev Drug Discov 2005; 4: 854–865. 16224456 10.1038/nrd1854

[bib36] Wannemuehler TJ, Manukyan MC, Brewster BD, Rouch J, Poynter JA, Wang Y et al. Advances in mesenchymal stem cell research in sepsis. J Surg Res 2012; 173: 113–126. 22225756 10.1016/j.jss.2011.09.053

[bib37] Gonzalez-Rey E, Anderson P, Gonzalez MA, Rico L, Buscher D, Delgado M. Human adult stem cells derived from adipose tissue protect against experimental colitis and sepsis. Gut 2009; 58: 929–939. 19136511 10.1136/gut.2008.168534

[bib38] Luo CJ, Zhang FJ, Zhang L, Geng YQ, Li QG, Hong Q et al. Mesenchymal stem cells ameliorate sepsis-associated acute kidney injury in mice. Shock 2014; 41: 123–129. 24169208 10.1097/SHK.0000000000000080

