# Supplementary material for: Mesenchymal stem cells in alleviating sepsis-induced mice cardiac dysfunction via inhibition of mTORC1-p70S6K signal pathway
Source: Cell Death Discov. 2017 Feb 27;3:16097–. doi: 10.1038/cddiscovery.2016.97 (PMC5327616; doi:10.1038/cddiscovery.2016.97)
Supplement: Supplementary Information [file cddiscovery201697-s1.pdf]

Supplemental Data

Identification of Raptor knockout

Cardiac-specific Raptor (regulatory associated protein of mTOR, complex 1) knockout (Raptor KO) mice were obtained from Rap^{loxP} mice crossed with alpha-MHC-Cre^{+} FVB/N background mice. Rap^{loxP} (stock number: 013188) and alpha-MHC-Cre^{+} (stock number: 011038) mice were acquired from the Jackson Laboratory. The genotype, possessed of Rap^{loxP} homozygote and alpha-MHC-Cre^{+}, were identified as the Jackson Laboratory`s genotyping protocols (Fig. S1A).

Furthermore, we evaluated the expression of myocardial Raptor by immunoblotting. The result showed that the expression of Raptor in Raptor KO group (0.08432±0.004826 vs 0.3560±0.03111, n=6, p<0.05, n=3) significantly decreased compared with WT mice (Fig.S1B). In addition, Raptor KO mice`s EF (57.25±3.746% vs 52.07±4.372%, p>0.05) and FS (35.20±3.298% vs 30.05±5.661%, p>0.05), evaluated by echocardiography, were no remarkable changes in comparison with WT mice (Fig.S1C).

Plasmid construction and adenoviral infection

RPTOR gene was obtained through NheI & BamHI E. coli from HEK293 cell and amplified with the following primer: 5’AACCCCGGTCCGGCTAGCGCCACCATGGAGTCGGAGATGCTGCAGT 3’ Rev: 5’ GCCAGATCCGCCGGATCCTCTGACACGCTTCTCCACCGAA 3’ with a Flag at the end. Preparation of competent cell and transforming was administrated as the 《Molecular Cloning》instructed. Expression was confirmed by PCR with the following primer For: CMV-MF: TCATTCTATTGGCTGAGCTGCG. EGFP-R: TCAGCTTGCCGTAGGTGGCAT (Fig S2A and S2B).

The viral packaging was administrated through a method which the recombination plasmid with gene of interest (CN1037) and skeleton adenoviral plasmid were cotransfected into HEK293 cell (Fig. S3A). Then, the virus was with a slight amplification. In brief, the recombinant vector was linearized and transfected

into low passage HEK293 cells. After 5 – 6 days, transfected cells were subjected to 3 freeze/thaw cycles and viruses were harvested (Fig. S3B).. Furthermore, the viruses were amplified and purified. Cells were pelleted and the supernatant was used to infect additional HEK293 cultures to expand and amplify the virus. Supernatant and cells were harvested from infected plates of HE K293 cells. Cells were then pelleted and the supernatant was loaded on top of a CsCl gradient and centrifuged at 22800 rpm 4℃ 25 hours. The viral band with a density of 1.30-1.40g/mL was extracted and transferred to the dialysis cassette, dialyzing overnight. Meanwhile, the buffer was changed one time. The adenovirus was stored in the -80. The titer of the virus was measured by performing a standard plaque assay (Fig. S3C).

Cytomix preparation

Macrophages(RAW264.7) (1 ×106/well) were seeded in 6-well plate, after 24-hour incubation, LPS (100 ng/mL) was then added to the lower chamber for 1 h, without /with (cytomix1/ cytomix2) BM-MSCs (2 ×105/well) seeded in the 6-well Transwell inserts (5 μm pore, Corning Incorporated, NY, USA) .（Fig.S4A） Then， the two type of medium in the lower chamber were collected and named cytomix1 and cytomix2, respectively, stored in -80℃.

Co-culture of H9c2 and BM-MSCs

Cardiomyocytes (H9c2) (1 ×106/well) were seeded in 6-well plate , and then knocked down or overexpressed Raptor with siRNA or adenovirus, after 24-hour incubation, the medium were replaced with cytomix1 or cytomix2 ， then co-cultured without or with BM-MSCs for 72h, and H9c2 lysate was collected for protein analyses(Fig.S4B). 9 groups were designed :1）Con 2）Cytomix 3) Cytomix+BMSCs 4)RPTOR(K.D) 5) RPTOR(K.D)+ Cytomix 6) RPTOR(K.D)+ Cytomix+BMSCs，7）RPTOR(O.E) 8) RPTOR(O.E)+ Cytomix 9) RPTOR(O.E)+ Cytomix+BMSCs

Differentiation potential of BM-MSCs.

Adipogenesis of BM-MSCs under the adipogenic differentiation conditions was detected by Oil red O staining. Osteogenesis was evaluated by Alizarin red S staining (scale bar, 20μm).
